# Single-Incision Laparoscopic Colectomy for Cancer: Short-Term Outcomes and Comparative Analysis

**DOI:** 10.1155/2013/283438

**Published:** 2013-05-19

**Authors:** Rodrigo Pedraza, Ali Aminian, Javier Nieto, Chadi Faraj, T. Bartley Pickron, Eric M. Haas

**Affiliations:** ^1^Division of Minimally Invasive Colon and Rectal Surgery, Department of Surgery, The University of Texas Medical School at Houston, Houston, TX, USA; ^2^Colorectal Surgical Associates, LLP, Ltd, Houston, TX 77054, USA

## Abstract

*Introduction*. Single-incision laparoscopic colectomy (SILC) is a viable and safe technique; however, there are no single-institution studies comparing outcomes of SILC for colon cancer with well-established minimally invasive techniques. We evaluated the short-term outcomes following SILC for cancer compared to a group of well-established minimally invasive techniques. *Methods*. Fifty consecutive patients who underwent SILC for colon cancer were compared to a control group composed of 50 cases of minimally invasive colectomies performed with either conventional multiport or hand-assisted laparoscopic technique. The groups were paired based on the type of procedure. Demographics, intraoperative, and postoperative outcomes were assessed. *Results*. With the exception of BMI, demographics were similar between both groups. Most of the procedures were right colectomies (*n* = 33) and anterior resections (*n* = 12). There were no significant differences in operative time (127.9 versus 126.7 min), conversions (0 versus 1), complications (14% versus 8%), length of stay (4.5 versus 4.0 days), readmissions (2% versus 2%), and reoperations (2% versus 2%). Oncological outcomes were also similar between groups. *Conclusions*. SILC is an oncologically sound alternative for the management of colon cancer and results in similar short-term outcomes as compared with well-established minimally invasive techniques.

## 1. Introduction

Minimally invasive colorectal surgery has been demonstrated to be a safe and efficacious approach for the surgical management of benign and malignant conditions [1–3]. Conventional multiport laparoscopy was the first utilized minimally invasive surgical modality for the management of colorectal diseases [4]. Thereafter, hand-assisted laparoscopic surgery was in part developed to overcome some of the technical challenges of conventional laparoscopic surgery [5]. These approaches require several incisions for port placement and specimen extraction, which may potentially results in complications such as extraperitoneal insufflation, bleeding, and internal organ injury [6]. In an attempt to progress with less invasive techniques and diminish potential complications, minimally invasive colorectal surgery is trending towards reduced-port modalities.

Single-incision laparoscopic colectomy (SILC) is an emerging surgical technique that has gained enthusiasm and interest in the field of colorectal surgery [7–9]. In this approach, the entirety of the procedure is performed through one incision, which would be otherwise required for specimen extraction, and it is potentially associated with reduction of port-site and incision-related morbidity, reduced postoperative pain, and improved cosmesis [7, 10, 11]. As compared to other minimally invasive techniques, SILC has also been shown to provide additional benefits such as lower surgical blood loss and quicker recovery [7, 9, 12]. The utility of SILC for the curative intent of colon cancer has yet to be evaluated in a single-institution case-control study. 

The aim of this study was to evaluate the short-term outcomes following SILC for the management of colon cancer and to compare these results to the established conventional laparoscopic and hand-assisted laparoscopic modalities. 

## 2. Methods

The data from this study was obtained from an Institutional Review Board approved database. From July 2009 to October 2011, a total of 167 patients underwent SILC for benign and malignant colorectal diseases in our practice, among which 50 had resection for adenocarcinoma of the colon. These 50 patients represent the SILC arm of the study and were paired based on the diagnosis of cancer and type of colectomy with the last 50 patients who had undergone minimally invasive colectomy with the utilization of either conventional multiport or hand-assisted laparoscopic techniques. The latter represents the second study arm (MIS group), and the selection of conventional laparoscopic colectomy (CLC) or hand-assisted laparoscopic colectomy (HALC) was depending on surgeon preference. The procedures were performed by one of two board certified colorectal surgeons with extensive experience in minimally invasive colorectal surgery (T. B. P. and E. M. H.). Demographic data including age, gender, body mass index (BMI), and history of previous abdominal surgery were analyzed. Intraoperative outcomes including estimated blood loss (EBL), operative time (OT), and conversion rate were assessed. Postoperative results were tabulated and analyzed following 30 days after discharge and included complication rate, length of stay (LOS), readmission rate, and reoperative intervention. Histopathologic characteristics including number of extracted lymph nodes, status of surgical margins, stage, and grade of tumor were assessed.

### 2.1. Surgical Technique

We have previously described our technique and port placement [8, 13–16]. The SILC procedures were performed with the utilization of one of two single-port devices: SILS Port Multiple Instrument Access Port (Covidien, Mansfield, MA) or GelPOINT Advanced Access Platform (Applied Medical, Rancho Santa Margarita, CA). A 30-degree 5 mm standard laparoscope and conventional nonarticulating laparoscopic instruments were used for all procedures. All cases were performed with a medial-to-lateral approach with early identification and high division of vascular structures. Other oncologic resection principles were maintained, such as minimized manipulation of the tumor, complete mobilization to reach appropriate margins, controlled release of pneumoperitoneum before removal of ports, and use of a wound protector for specimen extraction. 

### 2.2. Statistical Analysis

Continuous parameters are presented as the mean ± standard deviation, median, and range. Categorical data are expressed as percentage. Comparative analysis was performed with Student's *t*-test and chi-square test. *P* value < 0.05 was considered as a criterion of statistical significance.

## 3. Results

A total of 50 patients who underwent SILC for the management of colon adenocarcinoma were evaluated and compared to an MIS group comprised of 50, HALC (*n* = 37), and CLC (*n* = 13). On each arm the most common procedure was right hemicolectomy (*n* = 33), followed by rectosigmoid resection (*n* = 12), transverse colectomy (*n* = 2), left hemicolectomy (*n* = 2), and subtotal colectomy (*n* = 1). Demographic data are summarized in Table 1. There was no significant difference between SILC and the MIS group with regard to age (64.6 ± 12.4 years versus 66.3 ± 12.9 years, *P* = 0.49), gender (50% versus 54%, *P* = 0.69), ASA score (2.5 ± 0.7 versus 2.7 ± 0.6, *P* = 0.06), and history of prior abdominal surgery (48% versus 58%, *P* = 0.32). The BMI in SILC group was 27.2 ± 5.7 kg/m^2^, whereas in the MIS group was 31.0 ± 8.1 kg/m^2^, which resulted in significant difference (*P* = 0.007). 

With regard to intraoperative results, the mean OT was similar in both groups, 127 ± 37.5 min for SILC and 126.7 ± 63.6 min for the MIS group (*P* = 0.9). The EBL was lower in the SILC group (64.4 ± 64.7 cc versus 87.2 ± 89.2), but not statistically significant (*P* = 0.15). In the SILC group, there were no conversions to open surgery, whereas in the MIS group, there was only one conversion to laparotomy as a consequence of a large bulky tumor. Five cases of the SILC group, however, were converted to HALC for dense adhesions (*n* = 3), inability to maintain pneumoperitoneum in a morbidly obese patient with a BMI of 40 kg/m^2^ (*n* = 1), and the necessity for incision lengthening for specimen extraction (*n* = 1). There was one intraoperative complication in the SILC group and none in the MIS group. The intraoperative outcomes are presented in Table 2. 

The mean of extracted lymph nodes was 21 ± 8.4 (range: 12–49) and 19.2 ± 7.6 (range: 10–39) for SILC and MIS group, respectively, (*P* = 0.17). All surgical margins were negative for malignancy in both groups (Table 2). 

The mean LOS was 4.5 ± 3.7 days and 4.0 ± 1.7 days for SILC and the MIS groups, respectively, (*P* = 0.42). The postoperative complication rates were 14% and 8% for SILC and the MIS groups, respectively, (*P* = 0.34). In the SILC group, one patient required readmission whereas 2 patients were readmitted in the MIS group. There was one reoperation in the SILC group and two reoperations in the MIS group (Table 3). 

## 4. Discussion

Single-incision laparoscopic colectomy has been demonstrated to be a safe and feasible minimally invasive surgical modality for colon resections. In addition to the perceived cosmetic benefits, this technique is associated with reduced postoperative pain, the potential for quicker recovery, and shorter length of stay [7, 9–12]. Moreover, the SILC technique eliminates the use of peripheral ports potentially reducing the risk for port-site bleeding, hernia, infection, and tumor recurrence. Several case series have evaluated outcomes following SILC; however, only a few have compared SILC to other well-established minimally invasive techniques. To date, there are two randomized controlled trials (RCTs) comparing SILC to CLC for the management of colon cancer. The first study by Huscher et al. assessed outcomes of 16 patients on each arm [17], whereas the second study by Poon et al. evaluates outcomes of 25 patients on each arm [11]. In addition, a large retrospective study by Champagne et al. reported outcomes following SILC and CLC in a cohort of 165 patients on each arm [10]. This report consisted of a multicenter, multiple-surgeon study, with the potential for confounding secondary to different postoperative pathways and management. In the present study, we retrospectively evaluated outcomes of 50 patients following SILC for the management of colon cancer and compared outcomes to one of two well-established minimally invasive surgical approaches, HALC and CLC. The present study represents a single-institution experience, which minimizes confounding factors such as surgeon experience and variations among institutions. 

In the present study, we found that the OT was nearly identical in both groups. Similarly, Champagne et al. [10] reported near equal OT in both arms. Huscher et al. and Poon et al. reported longer OT for SILC as compared to CLC by 18 and 31 minutes, respectively; however, the differences did not reach statistical significance [11, 17]. Single-incision laparoscopic colectomy presents some technical challenges resulting from the coaxial instrumentation alignment including a reduced the visual field, increased internal and external instrument clashing, and diminished range of motion. Accordingly, one may anticipate an increased OT during SILC. We believe that, as experience is gained, many of the SILC limitations may be overcome by technical modifications such as the utilization of different length instrumentation, the “hand-over-fist” maneuver with the resulting triangulation of tissues, and the utilization of an inferior-to-superior approach for right hemicolectomy [15, 16]. These adjustments result in the reduction of the procedure length, thus equalizing the OT of SILC to that of other minimally invasive techniques. Furthermore, we believe that by eliminating peripheral ports, SILC provides a platform to streamline the steps of the procedure and to reduce extraneous maneuvers throughout. 

Conversion to laparotomy during minimally invasive colorectal surgery has been reported to be as high as 29%, and it has been associated with slow recovery and high postoperative morbidity [1, 18]. In our series, one case required conversion to open surgery and occurred in the MIS group and was due to difficult dissection and exposure in the setting of a large, bulky tumor. In the SILC group, although there were no conversions to open surgery, five cases required conversion to HALC. Despite the challenges of the SILC approach, our conversion rate to laparotomy is low, which is consistent with other SILC studies [10, 11, 17]. In challenging SILC cases, a minimally invasive platform may be maintained by the placement of additional ports or conversion to HALC [8]. The HALC technique has become our preferred modality for conversion, as it is readily available requiring only an extension of the incision. Furthermore, it offers the advantages of an enhanced exposure, blunt digital dissection, and the confidence provided by the hand-assistance, which is particularly beneficial early in the learning curve. Additionally, the HALC approach results in outcomes similar to those of other MIS techniques and improved as compared to open surgery and thus the patients attain the benefits of a minimally invasive platform and the enhanced recovery measures. 

In our practice, we now favor the single-incision approach as the MIS option for the majority of colon resections. Although morbid obesity may be a factor predicting conversion, it is not an absolute contraindication of SILC [8]. We have found, however, that for those with a BMI of 35 or greater, the SILC approach is less ideal and the benefits to the patient may not outweigh the technical challenges of the procedure. 

Reported data typically shows that the SILC approach results in nearly identical or shorter LOS, as compared to CLC [10]. In the present study, the mean LOS in the SILC group was slightly longer than that of the MIS group; yet this difference was not statistically significant. This difference may be attributed to an overall low number of cases, and thus a sampling error. Furthermore, we are comparing a relatively new procedure comprising the initial surgeons' experience to techniques in which we had performed over one hundred cases. 

In this series, the overall complication rate was 12% and was similar between the SILC and MIS groups. In the SILC group, the most common complication was wound infection (*n* = 2), followed by anastomotic leak (*n* = 1), para-anastomotic abscess (*n* = 1), prolonged postoperative ileus (*n* = 1), stroke (*n* = 1), and respiratory failure (*n* = 1). The patient with the anastomotic leak required reintervention during the same hospital stay whereas the patient with the abscess required readmission, and was successfully treated with percutaneous drainage and systemic antibiotic therapy. In the MIS group the most common complication was anastomotic leak (*n* = 2), followed by wound infection (*n* = 1), pelvic abscess (*n* = 1), and respiratory failure (*n* = 1). One patient with anastomotic leak was reoperated during the same hospital stay and the other case was readmitted for reintervention. The pelvic abscess required readmission and was managed with percutaneous drainage. These similar results between SILC and CLC with regard to postoperative complications are consistent with the reported literature [10, 11]. 

The pathological evaluation demonstrated that all MIS techniques result in oncologically sound outcomes. In all cases the specimen had tumor-free proximal and distal margins. There was a slight, nonsignificant, difference in the median number of lymph nodes harvested between the SILC and MIS groups, 19.5 and 17, respectively. This was in accordance with current colorectal cancer guidelines [19]. In order to accomplish suitable oncological outcomes during minimally invasive colectomy, some technical considerations have to be taken into account. We perform a technique in which the neoplastic lesion is not manipulated during the procedure, thus eliminating the potential for intraperitoneal tumor seeding. The high ligation of vascular pedicles is also performed so as to maximize lymph node extraction, in addition to the utilization of a wound protector for specimen extraction in order to eliminate tumor seeding at the extraction site. 

The main limitation of this study was relatively small sample size and is limited to short-term followup. We matched the SILC cases to a series of HALC and CLC cases to overcome the small sample size, which may negatively affect comparisons. Moreover, the SILC procedures represent the initial experience of the surgeons with the single-incision platform, whereas the MIS group consisted of procedures performed after hundreds of cases of HALC and CLC. Although this would have resulted in poor SILC outcomes, this learning curve discrepancy did not compromise the results following the SILC technique, which compared similarly to the MIS group.

Single-incision laparoscopic surgery is a safe and efficacious alternative MIS approach for the management of colonic malignancies when performed by an experienced surgeon. This technique results in similar short-term operative and oncological outcomes when compared to well-established laparoscopic approaches such as conventional multiport and hand-assisted laparoscopic surgery. 

## Figures and Tables

**Table 1:** Preoperative characteristics.

Parameters	SILC (*n* = 50)	MIS (*n* = 50)	*P* value
Age (years)	64.6 ± 12.4	66.3 ± 12.9	0.49
Gender (F, %)	25.0 (50%)	27.0 (54%)	0.69
BMI (kg/m^2^)	27.2 ± 5.7	31.0 ± 8.1	0.007
ASA score	2.5 ± 0.7	2.7 ± 0.6	0.06
Prior abdominal surgery (%)	24.0 (48%)	29.0 (58%)	0.32
Operative procedure			
Right colectomy	33 (66%)	33 (66%)	
Transverse colectomy	2 (4%)	2 (4%)	
Left colectomy	2 (4%)	2 (4%)	
High anterior resection	12 (24%)	12 (24%)	
Subtotal colectomy	1 (2%)	1 (2%)	

BMI: body mass index; ASA: American Society of Anesthesiologists; MIS: minimally invasive group composed of conventional multiport and hand-assisted laparoscopic surgery; SILC: single-incision colectomy group.

**Table 2:** Intraoperative and pathological data.

Parameters	SILC (*n* = 50)	MIS (*n* = 50)	*P* value
Operative time (min)	127.9 ± 37.6	126.7 ± 63.6	0.9
Estimated blood loss (cc)	64.4 ± 64.7	87.2 ± 89.8	0.15
Complications (%)	1.0 (2%)	0	1.0
Conversion to open surgery (%)	0	1.0 (2%)	0.31
Extracted lymph nodes	21.4 ± 8.4	19.2 ± 7.6	0.17
Positive margins	0	0	

MIS: minimally invasive group composed of conventional multiport and hand-assisted laparoscopic surgery; SILC: single-incision colectomy group.

**Table 3:** Postoperative outcomes.

Parameters	SILC (*n* = 50)	MIS (*n* = 50)	*P* value
Complication (%)	7.0 (14%)	4.0 (8%)	0.34
Length of stay (days)	4.5 ± 3.7	4.0 ± 1.7	0.42
Readmission (%)	1.0 (2%)	2.0 (4%)	0.56
Reoperation (%)	1.0 (2%)	2.0 (4%)	0.56

MIS: minimally invasive group composed of conventional multiport and hand-assisted laparoscopic surgery; SILC: single-incision colectomy group.
